# The Death of His Royal Highness the Prince Consort

**Published:** 1862-01

**Authors:** 


### THE DEATH OF HIS ROYAL HIGHNESS THE PRINCE CONSORT.

A GREAT GRIEF has fallen upon the nation from the death of His Royal Highness the PRINCE CONSORT. Such alleviation to our sorrow as may be derived from the reflection that whatever human knowledge and skill could do to ward off the fatal event, would be done by the physicians in attendance upon His Royal Highness (Sir James Clark, Sir Henry Holland, Dr. Watson, and Dr. Jenner), we have. Congestion of the lungs, supervening in the course of typhoid fever, was the immediate cause of death. On Sunday, the 8th of December, it was first made known that His Royal Highness was indisposed; the *Court Circular* of that day stating that he "had been confined to his apartments for the past week, suffering from a feverish cold, with pains in the limbs." On the Wednesday following, the first bulletin was issued, which stated that the Prince Consort was "suffering from fever, unattended by unfavourable circumstances, but likely, from its nature, to continue for some time." On Thursday it was stated that His Royal Highness had passed a quiet night, and that the symptoms had undergone little change. On Friday evening a bulletin was issued, announcing that His Royal Highness had "passed a restless night, and that the symptoms had assumed an unfavourable character during the day." This was the bulletin which first made known that the illness of the Prince Consort had become serious; but the uneasiness which the news gave rise to, when it became generally known in the kingdom on the following morning, was considerably relieved by the bulletin issued early on Saturday, and which stated that the illustrious patient had passed "a quieter night," and that there was "some mitigation in the severity of the symptoms." But in the course of Saturday evening, the Metropolis was painfully startled by a bulletin, dated 4·30 p.m., and which stated that the Prince Consort was "in a most critical state;" and on Sunday morning the nation was immeasurably shocked to learn, that at ten minutes before eleven o'clock on the preceding evening, the Prince Consort had expired.

Thus died at Windsor, on the 14th ult., this great and accomplished Prince—great in all that renders man most noble and most beloved, accomplished in all that gives man intellectual supremacy. The nation claims a large share in the mighty affliction which has fallen upon the Queen and the Royal Household. Prince Albert's name was a household word; his death is a household grief.

